# Development of Nanocomposite-Based Strain Sensor with Piezoelectric and Piezoresistive Properties

**DOI:** 10.3390/s18113789

**Published:** 2018-11-06

**Authors:** Mehdi Sanati, Allen Sandwell, Hamid Mostaghimi, Simon S. Park

**Affiliations:** Department of Mechanical and Manufacturing Engineering, University of Calgary, 2500 University Drive N.W., Calgary, AB T2N 1N4, Canada; mehdi.sanati@ucalgary.ca (M.S.); asandwel@ucalgary.ca (A.S.); hamid.mostaghimi1@ucalgary.ca (H.M.)

**Keywords:** nanocomposite, sensor, strain, piezoelectric, piezoresistive, sensor fusion

## Abstract

Sensors provide an interface between mechanical systems and the physical world. With the move towards Industry 4.0 and cyber-physical systems, demands for cost-effective sensors are rapidly increasing. Conventional sensors used for monitoring manufacturing processes are often bulky and need complex processes. In this study, a novel high-sensitive nanocomposite-based sensor is developed for measuring strain. The developed sensor is comprised of polyvinylidene fluoride (PVDF) as a piezoelectric polymer matrix, and embedded carbon nanotube (CNT) nanoparticles creating a conductive network. Exhibiting both piezoelectric and piezoresistive properties, the developed sensors are capable of strain measurement over a wide frequency band, including static and dynamic measurements. The piezoresistive and piezoelectric properties are fused to improve the overall sensitivity and frequency bandwidth of the sensor. To simulate the sensor, a 3D random walk model and a 2D finite element (FE) model are used to predict the electrical resistivity and the piezoelectric characteristics of the sensor, respectively. The developed models are verified with the experimental results. The developed nanocomposite sensors were employed for strain measurement of a cantilever beam under static load, impulse excitation, free and forced vibrations, collecting both piezoelectric and piezoresistive properties measurements. The obtained signals were fused and compared with those of a reference sensor. The results show that the sensor is capable of strain measurement in the range of 0–10 kHz, indicating its effectiveness at measuring both static and high frequency signals which is an important feature of the sensor.

## 1. Introduction

Sensors are important devices for monitoring the condition of the mechanical systems and manufacturing processes. They can convert an external stimulus into a measurable signal. The demand for development of new sensors and monitoring techniques continuously increases as the industry moves towards development of the fourth industrial revolution (Industry 4.0) and higher levels of automation for improving the quality of manufactured goods. Strain and force sensors have a broad range of applications including industrial automation, robotics, medical, and manufacturing applications. Different sensing systems based on piezoelectricity, strain gauges, capacitance, and lasers have been developed for force, strain and displacement measurements. The available sensors mostly have a complex manufacturing process or demand expensive parts and complex signal processing setups which may increase the cost and time of production. In addition, size and flexibility are other challenges associated with existing sensors. The existing strain gauges and piezoresistive sensors are good candidates for low frequency measurements but drift errors that accumulates over time is a problem of conventional strain gauges [1]. To overcome the challenges associated with existing sensing systems, a new low-cost and flexible polymeric nanocomposite-based sensing system is developed in this study. 

Polymer nanocomposites (PNCs) are considered a new class of materials, which are made of combined polymer and non-organic nano-scale fillers. Carbon nanotubes (CNTs) are considered great candidates for fillers since they provide remarkable mechanical [2], electrical [3] and thermal properties [4]. In addition, CNTs have a one-dimensional (1D) structure and high aspect ratios (around 100–1000) which make them unique materials for developing conductive PNCs compared to other metallic particles [5]. CNTs can be dispersed at a molecular scale inside a polymer matrix due to their size, where they have lengths ranging from nanometers to micrometers and diameters from 0.2 to 100 nm. They add substantial electrochemical and electromechanical properties to the nanocomposite samples and make them a unique option for sensor applications [6,7]. Electrical conductivity of PNCs can increase significantly using even low concentrations of CNTs [8]. A CNT-PNC becomes suitable for sensing applications when the CNT concentration reaches a region called the percolation threshold. Outside of this region there is no significant change in electrical conductivity with the PNC behaving as an insulator below the region and a conductor above it. Piezoresistive properties of the CNTs can be used for strain measurement, as it is shown that CNT–PNC based strain sensors can contain higher sensitivities compared to the conventional strain gauges [9]. However, there are some challenges associate with piezoresistive CNT–PNC based strain sensors, including dispersion of the CNTs within the polymer matrix and CNT alignment. Nanocomposite materials developed by CNTs and thermoplastic polymers provide unique properties such as high strength, and high resistance to wear and corrosion [10]. Different polymer matrixes have been used by researchers for fabricating CNT–PNC nanocomposites. Selection of the base polymer should be taken with especial care as it has a significant effect on the overall performance of the CNT–PNC nanocomposite sensor. The specific polymer matrix should be selected for the sensor that can process readily with the CNTs and is able to improve the electrical and electromechanical properties of the nanocomposite sensor. Different parameters, including the polarity, viscosity and degree of crystallization of the polymer, should be taken into consideration when choosing the polymer matrix [11,12,13].

Since the sensor developed in this study is designed to exhibit both piezoelectric and piezoresistive properties, the base polymer should be selected among those polymers that can provide a piezoelectric response in the CNT–PNC nanocomposite. Therefore, poly-(vinylidene fluoride) (PVDF) was selected as the polymer matrix in this research due to its excellent piezoelectric properties [14]. Also, it is reported by Kim et al. that the addition of CNTs into a PVDF polymer matrix allows for activation of piezoelectric properties at lower voltages compared to pure PVDF samples [15]. Moreover, it has been shown that adding multiwall CNTs (MWCNTs) into PVDF improves its electromechanical properties [16] and changes the polymer’s semi-crystalline structure from α phase to β phase [17]. 

Adding CNT nanoparticles into the PVDF polymer results in a nanocomposite sensor. The fabrication process used for developing the PVDF–CNT nanocomposite sensor has a significant effect on the electric properties and performance of the nanocomposite. A uniform dispersion and alignment of CNTs inside the polymer matrix are required to obtain desirable physical and mechanical properties of PNCs [18]. To mix CNTs with polymers, different techniques, including solution blending accompanying ultrasonication [19], and melt blending [20] have been used. These techniques can improve the dispersion of CNTs inside the polymer matrix. 

The main objective of this study is to develop a new sensor for strain and force measurement over a wide frequency range including both static and high-frequency dynamic excitations. The developed sensor aims to overcome the limitations and problems associated with existing sensors. These sensors are thin and flexible and can be mounted on any surface. Also, the behaviour of the nanocomposite sensor is studied through numerical modeling. The developed simulations are verified after comparing the obtained results with those of experiments. These sensors are mainly developed for machining processes applications; however, they can be used for other applications such as condition monitoring and structural health monitoring. 

The novelties of this study include combining the piezoelectric and piezoresistive properties in a single sensor to achieve high frequency bandwidth compared to commercially available sensors. Most of the existing sensors are only suited for measurement of static and low frequency signals, or dynamic and high frequency signals. Combining and fusing both the piezoelectric and piezoresistive signals of the nanocomposite sensor is a novel work developed to achieve a wider frequency bandwidth suitable for low and high frequency measurements in a single sensing package along with a higher sensitivity.

This paper is organized as follows: in Section 2 the fabrication process of the nanocomposite sensor using spray-coating technique is presented. The process for improving the piezoelectricity of the sensor, including stretching and poling, is then described. The experimental setup prepared for verifying the performance of the senor is elaborated afterwards. In Section 3, the developed piezoelectric and piezoresistive modelling and results are presented. The results of the developed sensor under static and dynamic excitations, including free vibration, impulse excitation, and forced vibration are then elaborated and discussed in Section 4. In addition, the sensor fusion method used for combining the piezoelectric and piezoresistive signals is presented along with the obtained results. Finally, the limitations associated with the developed sensor and assumptions made in the numerical modeling are discussed. Section 5 provides concluding remarks.

## 2. Fabrication Process and Experimental Setup

The fabrication process of the nanocomposite sensor, including the solution preparation and spray coating is presented. In addition, the process required for improving the piezoelectric *β* phase of the polymer matrix and subsequently increasing the piezoelectric coefficient of the sensor is discussed. Also, the experimental setups used for validation of the sensor are presented.

### 2.1. Fabrication of the Nanocomposite Sensor

**Figure 1:**
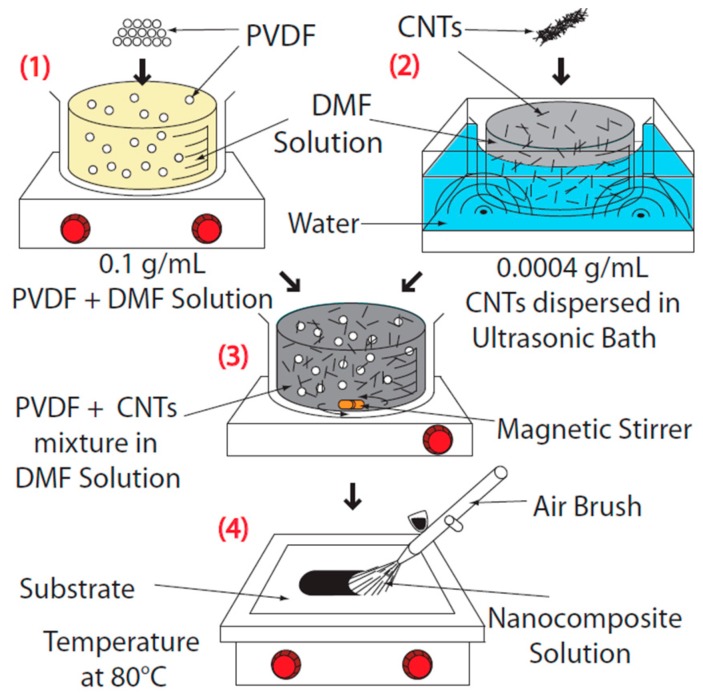
The schematic of the fabrication process.

**Figure 2:**
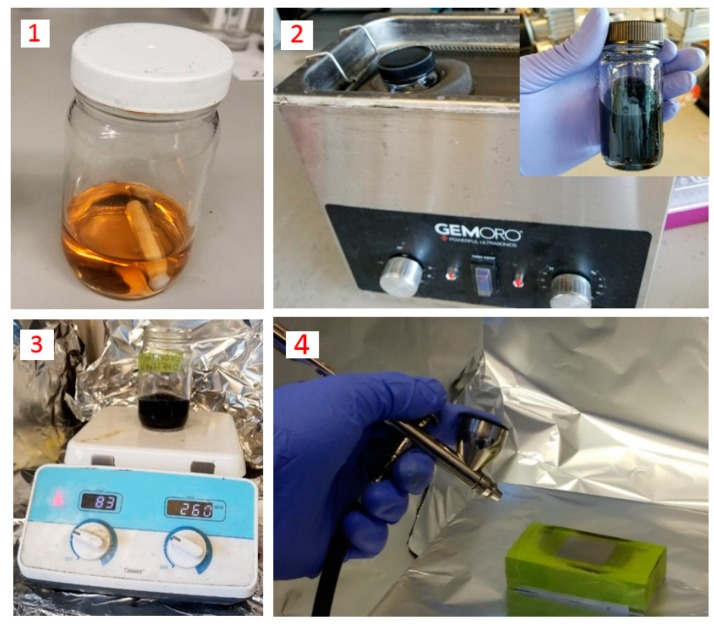
The fabrication process steps of the nanocomposite sensor.

Polyvinylidene fluoride (PVDF) is used as the polymer matrix of the developed sensor due to its high piezoelectricity. In addition, PVDF can form semi-crystalline structure (Ferroelectric) after poling. PVDF powder with the size of 100 nm was prepared from Sigma Aldrich. A solution of PVDF and N-N dimethylformamide (DMF) with 0.1 g/mL is stirred on the hot plate at 80 °C for 3 h until the polymer is fully dissolved. CNTs with 0.0004 g/mL concentration are separately mixed in DMF and sonicated at room temperature for 30 min to achieve a homogeneous dispersion of nanoparticles. The mixture is then added to the PVDF-DMF solution and stirred for 1 additional hour at room temperature. Once the mixture is prepared, a thin layer with 70 μm thickness is deposited using spray coating. The spray system used in this study is an air brush system connected to a compressed air supply. The nanocomposite mixture is poured inside the air brush system and fine droplets are deposited onto a glass substrate. The sprayed layer of the nanocomposite is heated at 80 °C till the solvent is fully evaporated and then the film is peeled off of the substrate. The schematic of the fabrication process is illustrated in Figure 1. The different steps associated with the fabrication process of the nanocomposite sensor is shown in Figure 2.

The CNT network inside the polymer matrix results in a conductive network and, therefore, provides piezoresistive properties needed for measurement of static and low frequencies loads and strains. However, it is still required to activate the piezoelectric properties of the PVDF polymer to measure higher frequencies with increased sensitivity. There are many ways of accomplishing this, but for this study two sequential processes of mechanical stretching and high-voltage poling are used to change the structure of the PVDF polymer and increase its piezoelectric properties. These processes are elaborated in the next subsection.

### 2.2. Sequential Stretching and Poling

**Figure 3:**
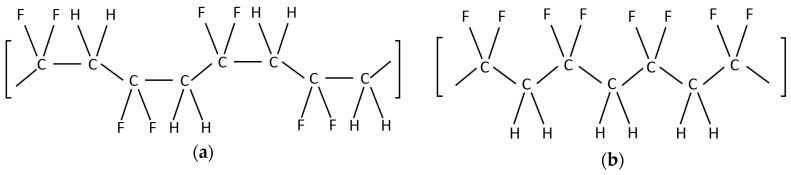
Schematic of polyvinylidene-fluoride (PVDF) structure (**a**) α phase and (**b**) β phase.

Polyvinylidene-fluoride (PVDF) is a semi-crystalline material consisting of four mainly conformations known as *α*, *β*, *γ*, and *δ* phase [21]. The C–F bonds of PVDF are polar and the highest amount of polarization can be obtained from the *β* phase where the all dipoles of the polymer are aligned in the same direction as shown in Figure 3. On the other hand, the *α* phase of PVDF orients the dipole moments in random directions, and consequently zero net-polarization is observed.

To improve the piezoelectricity of the prepared samples, the *β* crystallites of PVDF should increase and the existing *α* phase of PVDF should be converted to the *β* phase. There are different ways to promote this transition and improve the piezoelectric properties of the sensor, including mechanical stretching, electrical poling, solution casting, spin coating, and electro spinning [22]. Mechanical stretching, and electrical poling of the samples are utilized sequentially in this study for improving the *β* phase and subsequently the piezoelectricity of the sensor. It is presented in the literature that the sequential stretching and poling can result in improved piezoelectric properties in PVDF-based samples [23]. 

**Figure 4:**
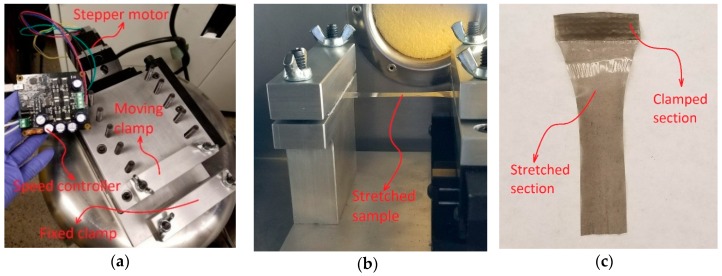
(**a**) Custom mechanical stretching device; (**b**) stretching of nanocomposite; (**c**) sample of the stretched nanocomposite film.

The prepared samples were first stretched mechanically at the temperature of 80 °C. It is shown that the transition from α phase to *β* phase is less effective at temperatures above 80 °C [24]. A custom device was built for stretching the nanocomposite samples as shown in Figure 4. The sample is held between a fixed and moving clamp. A stepper motor driver and linear stage is used to provide the movement. The speed of the moving clamp is adjusted using an electronic speed controller and the sample is stretched to 500% elongation. It has been shown that there is a direct relationship between the stretching ratio and transformation from α phase to *β* phase of PVDF [25]. Li et al. showed that the amount of *β* phase rapidly increases by increasing the stretching ratio up to about 300% elongation [25]. However, after this value, the *β* phase transition reaches a constant value and does not change considerably. Applying more stretching ratio, such as 500% in this study, results in a thinner film and subsequently allows a higher electric field during the poling process with the same voltage potential applied to the system. Therefore, alignment of the dipoles of the polymer occurs more efficiently and a higher piezoelectric coefficient is achieved.

Fourier transform infrared spectroscopy (FTIR) spectra of the sample before and after stretching provides insight about the structure of the PVDF polymer. FTIR allows the identification of different crystalline forms and to monitor the transition between different phases. Among different phases of the PVDF polymer, *γ* and *β* phases are similar in their polymer structure and can be identified by the absorbance peak at certain wavenumbers. For example, the band at 840 cm^−1^ is attributed to both *γ* and *β* phases, and this peak carries a dual signature of both phases [26]. However, the band at 1279 cm^−1^ is exclusively attributed to the *β* phase [26] and consequently monitoring the absorbance peak in this wavenumber can provide information about the *β* phase transition in the sample after stretching and poling. 

**Figure 5:**
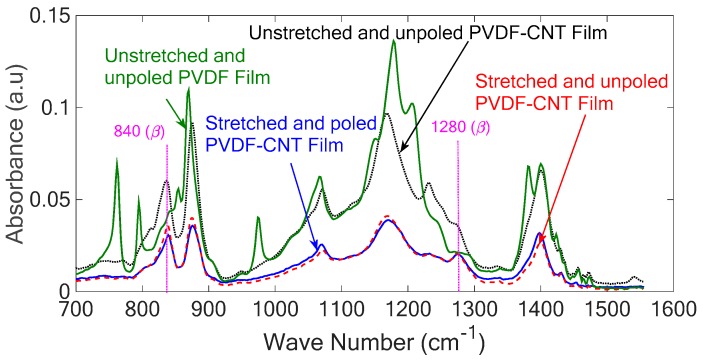
Fourier transform infrared spectroscopy (FTIR) of the PVDF and PVDF-carbon nanotube (CNT) samples before and after stretching.

The FTIR spectra of the prepared samples, i.e., pure PVDF, unstretched PVDF–CNT, stretched PVDF–CNT and polled PVDF–CNT, are presented in Figure 5. As can be seen, the unstretched PVDF sample contains mostly α phase due to the high absorbance peaks at 763, 795 and 974 cm^−1^, and almost no peak corresponding to *β* phases is observed which demonstrates that the polymer mainly consists of the non-polar *α* phase.

Gregorio and Cestari calculated the absorption coefficients, $K_{\alpha }$ and $K_{\beta }$, at the receptive wavenumbers 766 and 840 cm^−1^ after assuming that the FTIR absorption follows the Lambert–Beer law and presented Equation (1) for calculating the *β* phase of PVDF considering that the polymer is comprised of only *α* and *β* phases [27]:

(1) $$\beta =\frac{A_{\beta }}{\left(\frac{K_{\beta }}{K_{\alpha }}\right)A_{\alpha }+A_{\beta }}$$

where *β* represents the beta phase content, $A_{\alpha }$ and $A_{\beta }$ are the absorbance in wavenumbers 766 and 840 cm^−1^, respectively, and $K_{\alpha }$ and $K_{\beta }$ equal to $6.1\times 10^{4}$ and $7.7\times 10^{4}$ cm^2^mol^−1^, respectively.

Adding CNTs into PVDF show an increase in the absorbance at wavenumber 840 cm^−1^ corresponding to the *γ* and *β* phases. However, there is no considerable absorption peak at 888 and 1234 cm^−1^ corresponding to *γ* phase which implies that the polymer is mainly made of *α* and *β* phases. Based on Equation (1) adding CNTs into PVDF increases the *β* phase content from 32% to 78%. After stretching the *β* phase increases to 83% and the associated peaks at 840 and 1280 cm^−1^ became more pronounced, indicating an improvement of the polar *β* phase in the samples. In addition, the obtained results prove that reheating and poling of the samples after stretching do not have any remarkable effect on the *β* phase of the samples, but it is necessary to align the newly formed *β* phase crystallites in a single direction to improve the piezoelectricity of the sample.

To see the effect of stretching on the piezoelectricity of the prepared samples, the piezoelectric charge coefficient of the sample along the thickness direction is measured. This parameter is defined as the ratio of electric charges per unit area generated in response to an applied force. Considering that the thickness of the nanocomposite is negligible, and the poling occurs in the thickness direction, i.e., in the z direction, the only charge coefficient component which needs to be measured is *d*_33_. This component is defined as the ratio of the charge density and the applied mechanical force to the sample as shown in Equation (2).

(2) $$d_{33}=\frac{dq}{dF}$$

where *dq* is the charge density and *dF* is the applied mechanical force.

**Figure 6:**
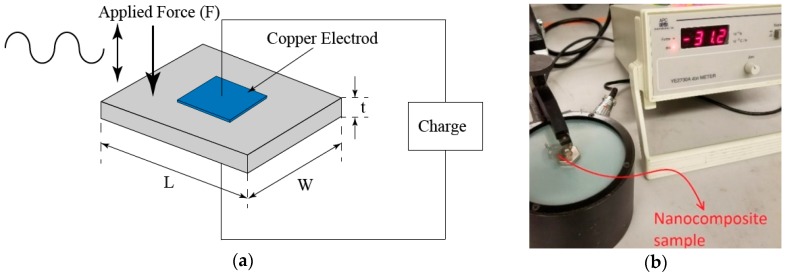
(**a**) Schematic of the piezoelectric coefficient (*d*_33_) measurement; (**b**) measured piezoelectric coefficient (*d*_33_) of the stretched sample after poling.

The above equation shows that there is a linear relationship between the applied force to the piezoelectric materials and the electric charge generated in the sample. A schematic detailing the process of measuring the piezoelectric coefficient (*d*_33_) is shown in Figure 6a.

A wide range *d*_33_ tester (APCI YE2730A *d*_33_ meter) was used to measure the piezoelectric coefficient of the sample. The measured value for the stretched sample shows that there is no improvement in the piezoelectric coefficient of the sample after stretching although the *β* phase content improved significantly compared to the pure unstretched PVDF. The reason behind this is the fact that the dipoles associated with the *β* phase are randomly arranged inside the polymer and consequently the net piezoelectric coefficient is almost zero. 

The problem regarding random orientation of dipoles and aligning them in a single direction can be solved by including a high voltage poling process. There are usually two main methods for poling the samples, namely contact poling and corona poling. Issues associated with contact poling approach include arcing problems at high voltages, damaging the sample, and disrupting the poling process. Therefore, the corona poling method is chosen, although this approach is more complex. The most important advantage of the corona poling approach over the contact poling method is the fact that arcing and short circuits are minimized during the process and the electric filed is more uniform. In addition, the poling process will not be disrupted even if there is arcing problem in the system since only few charges will pass through the resulting damage and the rest will keep the electric field and poling process. 

**Figure 7:**
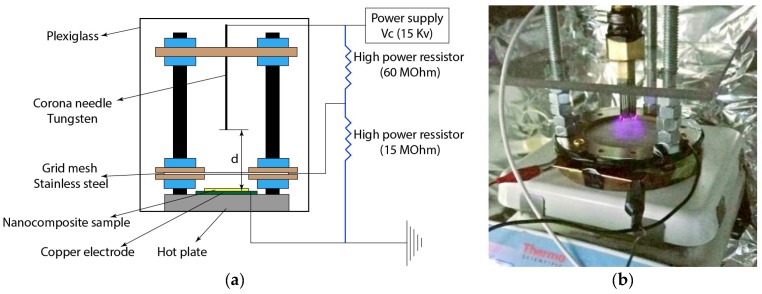
(**a**) Schematic of the corona poling; (**b**) Actual image of the corona poling process.

A schematic of the corona poling setup used in this study along with a figure of an actual poling process are shown in Figure 7. Parameter *d* in this figure shows the distance between the needle and the copper electrode connected to the ground. As can be seen, a copper electrode connected to the ground is placed on a hot plate and the nanocomposite sample is mounted on top of it. Also, a corona needle connected to a high-voltage power supply (15 kV) is placed above the sample and the grid mesh. A metallic grid mesh is used to generate a uniform electric field during poling. A voltage divider made of high power resistors are built to create a voltage of 3 kV applied to the grid mesh. During the process, the air inside the enclosure becomes ionized when the corona needle is connected to the high voltage power supply. The ionized particles move through the grid mesh and towards the ground plane and deposit on the surface of the nanocomposite sample. The resulting electric field provides alignment of the *β* phase crystallites in a single direction, which is the thickness direction in this study.

The piezoelectric coefficient of the nanocomposite sample after poling was measured using the *d*_33_ tester where an improvement of −1.6 Pc/N to −31.2 Pc/N was observed, as shown in Figure 6b.

With a completed sensor, different static and dynamic tests are performed to verify the performance and accuracy of the nanocomposite sensor in strain measurement. The experimental setups used in this study are discussed in the next subsection.

### 2.3. Experimental Setup

**Figure 8:**
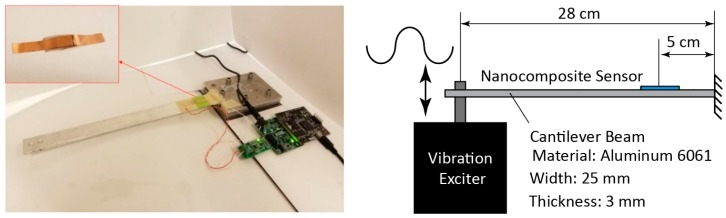
The experimental setup used in this study.

The performance of the developed sensor is verified through different experiments. The experimental setup used in this study is shown in Figure 8. As depicted in the figure, the developed sensor is mounted on an aluminum cantilever. Both sides of the fabricated nanocomposite film are connected to copper electrodes so the generated charge and resistivity changes of the sensor under strain are captured.

The nanocomposite sensor is attached to the cantilever beam using double-sided tape. The sensor is mounted near the clamped end of the cantilever where the predicted strain is highest. A charge amplifier system and a voltage divider are used for the piezoelectric and piezoresistive sensors, respectively. 

A vibration exciter (B&K Type 4808) is used to apply excitations to the beam for forced vibration tests; but it is removed for free vibration and static tests. A commercial metal foil strain gauge (Omega SGD-2/350-LY11) and an accelerometer (Kistler type 8778A500) are used for verifying the performance of the nanocomposite sensor in both static and dynamic tests. The applied force to the cantilever causes elastic strain in the beam which in turn results in generating electric charge and changes in the resistivity of the sensor that can be captured using a data acquisition system. For the impulse excitation test, the nanocomposite sensor is placed on a beam and the frequency response function (FRF) of the structure is measured experimentally. A piezoelectric hammer (PCB 084A17) is employed for applying an impulse excitation to the beam structure.

In the next section, two numerical modeling approaches are introduced for studying the piezoelectric and piezoresistive properties of the developed nanocomposite-based sensor. The developed finite element (FE) simulations are verified after comparing the numerical and experimental results.

## 3. Numerical Modeling

To further understand the behaviour of the sensing mechanism and the effect of strain on the electro-mechanical properties, numerical models are developed. As the sensor exhibits both piezoresistive and piezoelectric properties, two separate studies are performed to analyze the contributing effects of each phenomenon.

### 3.1. Piezoresistive Modeling

**Figure 9:**
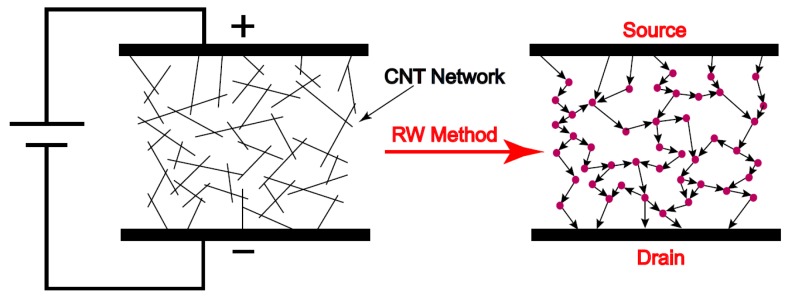
Schematic of random walk model approach for calculating the resistivity of a CNT network.

To predict the piezoresistive behaviour of the CNT-based nanocomposites, a 3D model is developed based on random walks on a finite network of CNTs [28,29]. In this model, the random walkers are simulated by electrical particles which are inserted into the CNT network starting at the source which has the highest electrical potential. They then move through the CNT network towards the nodes with the lowest potential called the drain. The electrical particles choose the pathways in the circuit depending on probability. Each walker is considered in the simulation and then the current and voltage of each node are calculated based on the paths taken by the particles. The total electrical current passing through the CNT network can be obtained and, when combined with the known source-drain potential, results in a value for the electrical resistivity of the network. The schematic of Random Walk model simulation for calculating the resistivity of a CNT network is shown in Figure 9.

In CNT-PNC nanocomposites, CNTs are in contact with each other through either direct contact or electron tunneling. Therefore, the resistance of the CNT nanocomposites mainly depends on the intrinsic resistance of the CNTs (*R_CNT_*) and the tunneling resistance (*R_tun_*) which is higher than the direct CNT resistance [30]. The tunneling resistance is usually observed when the insulating layer of the polymer is thin. 

**Figure 10:**
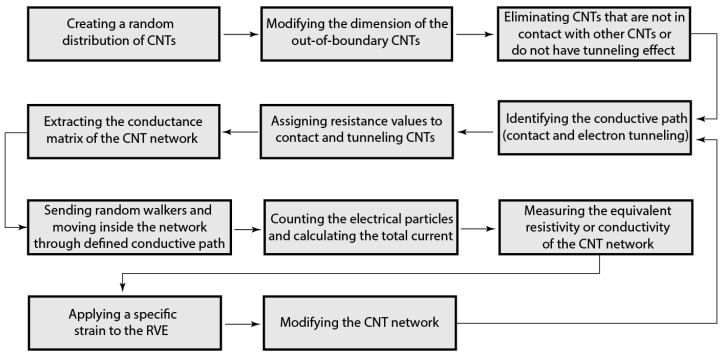
Process modeling of a piezoresistive CNT network using random walks method.

Different steps of the modelling process used in this study are presented in Figure 10. The FE simulation includes generating the representative volume element (RVE) using randomly distributing CNTs with variable lengths, determining the conductive pathways in the CNT network through identifying CNT–CNT contacts and tunneling distances, and finally forming the resistive network and calculating the resistivity of the CNT network.

The first step for modelling the CNT network is creating the RVE which represents the random distribution of CNTs inside a polymer matrix. A random distribution of CNTs with different lengths are generated using different parameters of the CNTs and the RVE as given in Equations (3) and (4):

(3) $$L_{CNT}=L_{M}+\left(0.3\times L_{M}\right)\times rand$$

(4) $$CNT_{num}=4\times Vol\times \left(\frac{L_{RVE}^{3}}{L_{M}\times \pi \times D^{2}}\right)$$

where *L_M_*, *L_CNT_* and *rand* are the mean length of all CNTs, the length of each CNT inside the RVE, and random numbers between −1 and 1, respectively. Also, *CNT_num_* is the number of the CNTs inside the RVE, *Vol* represents the CNT volume percentage, *L_RVE_* is the length of the RVE cube, and *D* is the diameter of the CNTs. The random distribution of CNTs provides an anisotropic and disordered network of CNTs. The ratio of the length of the RVE to the mean length of the CNTs ranges between 1 and 30 [31] and a ratio equal to 3.3 was chosen in this study since it is suggested as a safe ratio in literatures [31,32] and dramatically reduces the computational time. The length of the RVE cube was selected 1 μm × 1 μm × 1 μm which is large enough to represent the nanocomposite’s morphology with reasonable accuracy. Also, the mean length and diameter of CNTs were considered 0.3 μm and 20 nm, respectively. 

**Figure 11:**
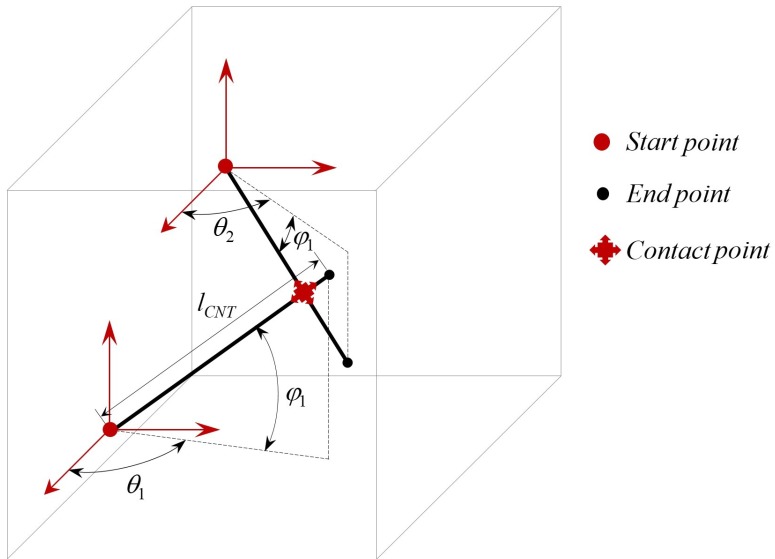
A representation of CNT networks with random selection of the start points, length and angles of CNTs.

After picking different random values for the lengths of the CNT network, different random points are selected as starting points for generating the CNT network inside the RVE. The end point of each CNT is defined using the length of each CNT and random selection of angles as given in Equation (5):

(5) $$x_{end}=x_{start}+L_{CNT}\cos\left(\theta \right)\;y_{end}=y_{start}+L_{CNT}\sin\left(\theta \right)\cos\left(\varphi \right)\;z_{end}=z_{start}+L_{CNT}\sin\left(\theta \right)\sin\left(\varphi \right)$$

where *φ* is the azimuthal angle, and *θ* represents the polar angle as shown in Figure 11.

In the next step, the generated CNTs that pass the boundaries of the RVE are identified and the out-of-boundary portion of the CNTs are cut to make sure all generated CNTs stay inside the RVE. In addition, the developed model eliminates the CNTs that are not connected to other CNTs or the electrodes. The CNTs that have a distance less than tunneling distance (1.8 nm [30,31]) with their surrounding CNTs are not eliminated since the electrons and random walkers can travel between them and create a conductive path. 

The updated CNT network is now used for calculating the resistivity of the nanocomposite and investigating the effect of strain on the piezoresistive effect. The conductance values of each contact and electron tunneling inside the CNTs network, which are reciprocals of resistance values, are calculated and stored in a conductance matrix which is an *N* × *N* matrix where *N* is the number of CNTs after updating. The CNT contact resistance is a function of the geometry and the electrical conductivity of the CNTs and is calculated using Equation (6) [33]:

(6) $$R_{CNT}=\frac{4L_{CNT}}{\pi \sigma_{CNT}D^{2}}$$

where *R_CNT_*, *L_CNT_* and *D* are the contact resistance, length of each CNT, and diameter of CNTs and $\sigma_{CNT}$ is the electrical conductivity of CNTs (5000 S/m).

The tunneling resistances can be obtained using Equation (7) [33]:

(7) $$R_{tun}=\frac{h}{2e^{2}}\frac{1}{M\tau_{p}}$$

where *R_tun_* is the tunneling resistance, *h* is the Plank’s constant (2πℏ), *e* is the electron charge ($\frac{h}{2e^{2}}\approx 12.9054\;k\Omega )$, *M* is the number of conduction channels, and $\tau_{p}$ is the transmission probability of an electron to tunnel between CNTs that can be obtained using the Schrodinger equation developed previously [33]. Contact resistance (Equation (6)) is considered for those CNTs which overlap or are in contact with each other while the tunneling resistance (Equation (7)) is considered for those CNTs that have a gap distance less than tunneling distance.

After extracting the conductance matrix from the obtained resistance values, the nodes connected to the source and drain are identified. Based on the method developed by Doyle and Snell, the electrical particles and random walkers are imported into the CNT network from the source nodes and move from one node to another through the conductive path till they finally reach the drain and leave the circuit [34]. The random walkers choose the path to move from source to drain based on the probability of each path inside the RVE. The probability of choosing node *j* when the electrical particle is on node *i* (*P_ij_*) can be calculated using the conductance of the path between *i* and *j* and the overall conductance of the whole network as presented in Equation (8):

(8) $$P_{ij}=\frac{G_{ij}}{\sum_{i=1}^{n}G_{ij}}$$

where *G_i__j_* is the conductance value of the CNT path between node *i* and *j*, and *n* is the number of nodes connected to node *i* in the CNT network through either direct contact or tunneling. 

An electrical potential of 1 V is applied across the source and drain nodes and the voltage at any node *i* of the network (*V_i_*) can be obtained using Equation (9). Voltage *V_i_* is a function of voltage *j* which is in contact with point *i* and the probability that a walker might move from point *i* to point *j*, i.e.,

(9) $$V_{i}=\underset{j}{\sum }P_{ij}V_{j}$$

To calculate the current *I_ij_* along a path from *i* to *j*, it is assumed that a walker might pass several times along the path from *i* to *j*, and in the opposite direction from *j* to *i*. Therefore, it is assumed that current *I_ij_* is proportional to the net number of movement along the path from *i* to *j*, where the movement from *j* to *i* is considered as negative [34]. Then,

(10) $$I_{ij}=\frac{\left(V_{i}-V_{j}\right)}{R_{ij}}$$

A random walker starts from a node on the source and pass a conductive path to reach the drain but if it returns to the source before reaching the drain, it keeps going. Finally, the total voltage between the source and the drain along with the total current given in Equation (11) are used to calculate the equivalent resistance of the CNT network as presented in Equation (12):

(11) $$I_{total}=\overset{n}{\underset{j=2}{\sum }}I_{1j}$$

(12) $$R_{eq}=\frac{V_{total}}{I_{total}}$$

where *n* is the number of CNTs connected to the source electrode. 

Changes in geometry of nanocomposites due to applied strains may result in contraction or expansion of the CNT network. In case of contraction of the network, number of connections between CNTs may increase which in turn results in less resistivity in the nanocomposite. Conversely, positive strain will decrease the number of CNT connections and, therefore, higher resistivity is achieved. 

**Figure 12:**
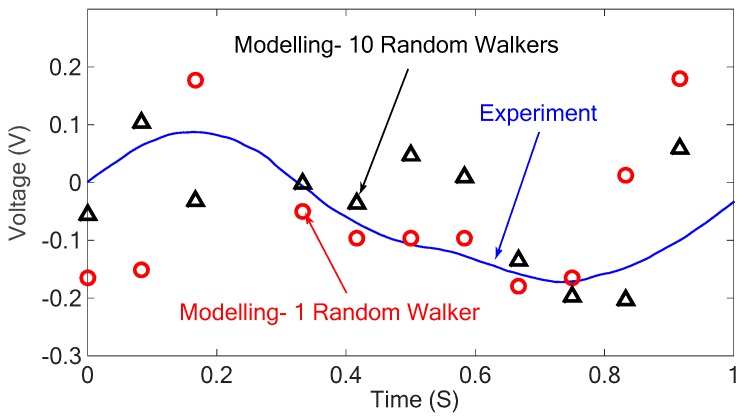
Piezoresistive results of the nanocomposite obtained from simulation and experiments.

To investigate the piezoresistive effect of the CNT network, strains are applied to the RVE in a particular direction and the changes in the resistivity of the system are calculated. Figure 12 shows the numerical and experimental results of the piezoresistive effect of the nanocomposite under different strains applied harmonically to the sensor.

As can be seen in the above figure, the numerical results for the resistivity of the nanocomposite under different strains follow a similar trend with the experimental results. Also, it is observed that the developed modeling approach results in higher resistance of nanocomposite when positive strain (tension) is applied to the system compared to when negative strain (compression) is applied to the nanocomposite as was expected. It can be seen in the obtained results that using more random walkers result in more accurate results; however, it increases the processing time significantly. The standard deviation value calculated for the simulation with 10 random walkers ($\sigma^{2}=0.0877$) is much lower than that of the simulation with 1 random walker ($\sigma^{2}=0.1302$). The piezoelectric properties of the nanocomposite are studied numerically in the next section and the discussed approach is verified with the experimental results.

### 3.2. Piezoelectric Modeling

The piezoelectric coefficient of the nanocomposites is predicted using a 2D Monte Carlo simulation. The model used in this study is based on a study conducted by Koenck et al. [35], in which randomness in nanotube size, location and orientation were incorporated in the determination of polymer nanocomposite piezoelectric parameters. The piezoelectric properties of nanocomposites can be predicted using a finite element approach as given in Equation (13) [35]:

(13) $$\left[\begin{array}{cc} K_{uu} & K_{u\varphi }\\ {K^{T}}_{u\varphi } & K_{\varphi \varphi } \end{array}\right]\left[\begin{array}{c} U\\ \Phi \end{array}\right]=\left[\begin{array}{c} F\\ Q \end{array}\right]$$

where *K_uu_* and $K_{\varphi \varphi }$ are the elastic stiffness matrix and dielectric stiffness matrix, respectively, $K_{u\varphi }$ is the piezoelectric coupling matrix, *U* and Φ are nodal displacements and nodal electrical potential, respectively, *F* is the nodal mechanical force, and *Q* is the nodal electrical charge. The model follows a three-step process to incorporate both the properties of the carbon nanotubes and surrounding polymer. In steps one and two, the elastic stiffness, piezoelectric coupling and dielectric stiffness matrices are calculated for the carbon nanotubes and polymer separately and in the third step, the effective piezoelectric parameters are computed using Equation (14):

(14) $$K_{eff}^{ele}=K_{m}^{ele}+\overset{n}{\underset{i=1}{\sum }}K_{nt}^{i}$$

where *K_eff_* represents the effective stiffness matrix for a single element, *K_m_* is the stiffness matrix of the polymer in the element, and *K_nt_* is the sum of all the stiffness matrices of all the nanotubes existing in the element. The following shows how to find the matrices in Equations (13) and (14) associated with both polymer element and embedded CNTs. 

#### 3.2.1. Calculation of Polymer Piezoelectric Parameters

The effective matrices in Equation (13) can be obtained by considering the properties of the polymer and the CNTs as given in Equation (14). The stiffness, piezoelectric coupling and dielectric stiffness matrices can be obtained using the formulations given in Equation (15):

(15) $$\begin{array}{c} K_{uu}=\int B_{u}^{T}CB_{u}dV\\ K_{u\varphi }=\int B_{u}^{T}CB_{\varphi }dV\\ K_{\varphi \varphi }=-\int B_{\varphi }^{T}CB_{\varphi }dV \end{array}$$

where *B_u_* is the strain and displacement and $B_{\varphi }$ is associated with the electrical field and potential. There is a direct relationship between the strain of an element and the displacement as given in Equation (16):

(16) S=Bu

where *S* is the strain and *u* is the displacement. Equation (17) gives a method for approximating the displacement using a series of shape functions *N* within each element:

(17) $$u=N_{u}U$$

Combining Equations (16) and (17) yields:

(18) $$S=BN_{u}U=B_{u}U$$

In the analysis, a four-node quadrilateral element with three degrees of freedom is chosen. In this case four shape factors, *N*_1_ … *N*_4_, per element are used and their partial derivatives are taken with respect to local coordinates *r* and *s*. *N_u_* and *B_u_* are then assembled as:

(19) $$N_{u}=\left[\begin{array}{cc} \begin{array}{ccc} N_{1} & 0 & \begin{array}{ccc} N_{2} & 0 & N_{3} \end{array} \end{array} & \begin{array}{ccc} 0 & N_{4} & 0 \end{array}\\ \begin{array}{ccc} 0 & N_{1} & \begin{array}{ccc} 0 & N_{2} & 0 \end{array} \end{array} & \begin{array}{ccc} N_{3} & 0 & N_{4} \end{array} \end{array}\right]$$

(20) $$B_{u}=\left[\begin{array}{ccc} \frac{\partial N_{1}}{\partial r} & 0 & \frac{\partial N_{2}}{\partial r}\\ 0 & \frac{\partial N_{1}}{\partial s} & 0\\ \frac{\partial N_{1}}{\partial s} & \frac{\partial N_{1}}{\partial r} & \frac{\partial N_{2}}{\partial s} \end{array}\begin{array}{ccc} 0 & \frac{\partial N_{3}}{\partial r} & 0\\ \frac{\partial N_{2}}{\partial s} & 0 & \frac{\partial N_{3}}{\partial s}\\ \frac{\partial N_{2}}{\partial r} & \frac{\partial N_{3}}{\partial s} & \frac{\partial N_{3}}{\partial r} \end{array}\begin{array}{cc} \frac{\partial N_{4}}{\partial r} & 0\\ 0 & \frac{\partial N_{4}}{\partial s}\\ \frac{\partial N_{4}}{\partial s} & \frac{\partial N_{4}}{\partial r} \end{array}\right]$$

where shape factors are given by:

(21) $$N_{1}=\frac{1}{4}\left(1-r\right)\left(1-s\right),\;N_{2}=\frac{1}{4}\left(1+r\right)\left(1-s\right)\;N_{3}=\frac{1}{4}\left(1+r\right)\left(1+s\right),\;N_{4}=\frac{1}{4}\left(1-r\right)\left(1+s\right)$$

The presented model is a two-dimensional (2*D*) square element, therefore,

(22) $$r=s=\pm \frac{1}{\sqrt{3}}$$

To map the local coordinates of each node to the global coordinates *x* and *y*, the Jacobian matrix presented in Equation (23) is used:

(23) $$J_{a}=\left[\begin{array}{cc} \frac{dx}{dr} & \frac{dy}{dr}\\ \frac{dx}{ds} & \frac{dy}{ds} \end{array}\right]$$

A similar approach can be used for determining *B_φ_*. To solve the volume integrals and determine the matrices in Equation (15), Gauss quadrature is employed. Therefore, Equation (15) is reformulated as:

(24) $$K_{m}^{ele}=\overset{n}{\underset{i=1}{\sum }}\overset{n}{\underset{j=1}{\sum }}B_{u}^{T}CB_{u}\|J_{a}\left(r,s\right)\|$$

This equation represents the elastic stiffness matrix of the polymer element. The piezoelectric coupling and dielectric stiffness matrixes associated with the polymer element can be obtained in a similar approach. Similarly, the corresponding matrixes need to be calculated for the embedded CNTs in the element as presented in the next subsection.

#### 3.2.2. Calculation of Carbon Nanotube (CNT) Piezoelectric Parameters

**Figure 13:**
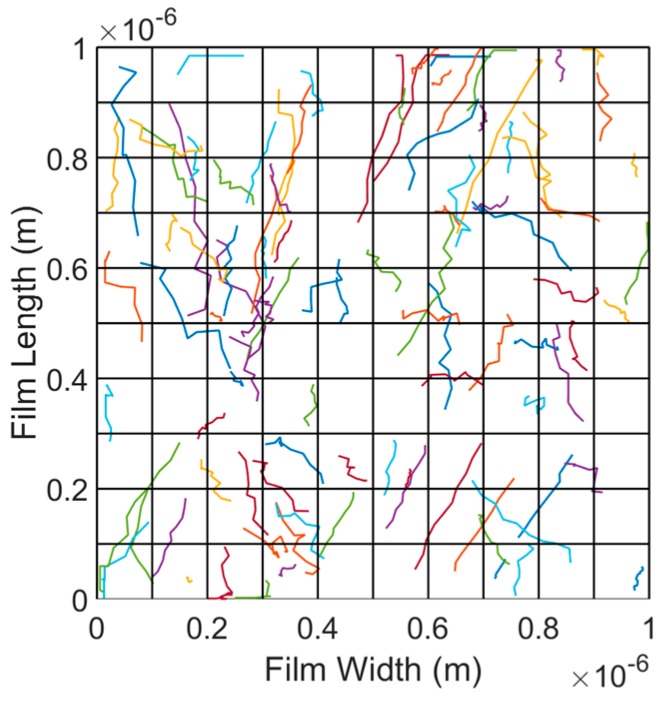
Embedded fibre method grid mesh with element partitioning.

In the modelling of the carbon nanotubes, the embedded fibre finite element (FE) method, first proposed by Esteva et al. [36] is used. In this method, an RVE populated with carbon nanotubes, in the desired concentration and orientation, is discretized into multiple elements. For the finite element model to function properly, each carbon nanotube crossing between elements must be partitioned. This is accomplished by use of the Liang-Barsky algorithm wherein each CNT is described parametrically:

(25) $$\begin{array}{c} x=x_{1}+\left(x_{2}-x_{1}\right)t\\ y=y_{1}+\left(y_{2}-y_{1}\right)t \end{array}$$

where (*x*_1_,*y*_1_) and (*x*_2_,*y*_2_) are the start and end points of the carbon nanotube, respectively, and *t* is any value between 0 and 1. The overall number of CNT lines in each element is determined after applying the Liang-Barsky algorithm to each CNT line with respect to each FE element. CNTs cross multiple elements when a denser mesh is used in the simulation; therefore, they are divided to multiple sections without affecting their properties as previously proved [36]. The resulting grid mesh of carbon nanotubes is shown in Figure 13.

To determine the piezoelectric parameters of the carbon nanotubes, each nanotube is approximated as a line element which results in efficient computation and sufficient accuracy [37]. Natural coordinates of the CNTs are used to determine the corresponding shape functions. To do so, the transformation matrix is defined as:

(26) $$T=\left[\begin{array}{c} N_{u}\left(\xi_{i},\;\eta_{i}\right)\\ N_{u}\left(\xi_{j},\;\eta_{j}\right) \end{array}\right]$$

where *i* and *j* represent the start and end points of the CNTs and each *N_u_* is a shape factor similar in form to Equation (20), and *r* and *s* are the coordinates of each carbon nanotube. The stiffness of each CNT within the element can be obtained using the following transformation matrix [35].

(27) $$K_{uu}^{nt}=T^{T}kT\;k=\frac{\left(E_{f}-E_{m}\right)A}{l}\left[\begin{array}{cc} \begin{array}{cc} \cos^{2}\alpha & \cos\alpha \sin\alpha \\ \cos\alpha \sin\alpha & \sin^{2}\alpha \end{array} & \begin{array}{cc} -\cos^{2}\alpha & -\cos\alpha \sin\alpha \\ -\cos\alpha \sin\alpha & -\sin^{2}\alpha \end{array}\\ \begin{array}{cc} -\cos^{2}\alpha & -\cos\alpha \sin\alpha \\ -\cos\alpha \sin\alpha & -\sin^{2}\alpha \end{array} & \begin{array}{cc} \cos^{2}\alpha & \cos\alpha \sin\alpha \\ \cos\alpha \sin\alpha & \sin^{2}\alpha \end{array} \end{array}\right]$$

where *E_f_*, *A* and *l* are the fibre’s Young’s modulus, cross-sectional area and length, respectively; and *E_m_* is the Young’s modulus of the polymer matrix material. The angle of the nanotube segment is given by *α*. The stiffness of all CNTs embedded inside the element can be found by a similar procedure as above. Then, the effective stiffness matrix in Equation (14) may be calculated.

As carbon nanotubes are not piezoelectric materials, the piezoelectric coupling matrix is 0. The dielectric stiffness matrix is formulated as follows [35]:

(28) $$K_{\varphi \varphi }^{nt}=\frac{-\varepsilon_{nt}A}{l}\left[\begin{array}{cc} 1 & -1\\ -1 & 1 \end{array}\right]$$

where *ε_nt_* is the dielectric constant of the CNTs. 

After computing the matrices for each embedded CNT in the element, the effective matrices of a single element can be obtained. Finally, the boundary conditions are applied to the RVE and the effective piezoelectric coefficient of the nanocomposite can be then calculated.

#### 3.2.3. Finite Element (FE) Model Solution

The material properties of MWCNT/PVDF nanocomposites were used for computing the effective piezoelectric charge coefficient, *d*_33_, of the composite material using the described FE simulation. The simulation boundary conditions include a fixed relation along the bottom edge with a force of 10 N applied on the top edge. In addition, no electrical potential was applied to both edges and, therefore, an electrical field was induced in the nanocomposite material due to the piezoelectric nature of the PVDF polymer. The model parameters used for the simulation are shown in Table 1 [10]. 

To verify the accuracy of the developed Monte Carlo model, an FE simulation was performed on about 100 different microstructures with the RVE geometry of $1\times 1\;{\mu m}^{2}$. The piezoelectric charge coefficient (*d*_33_) is extracted by averaging the results over 100 randomly generated RVEs and the obtained result is compared with that of the experiments as shown in Table 2.

**Figure 14:**
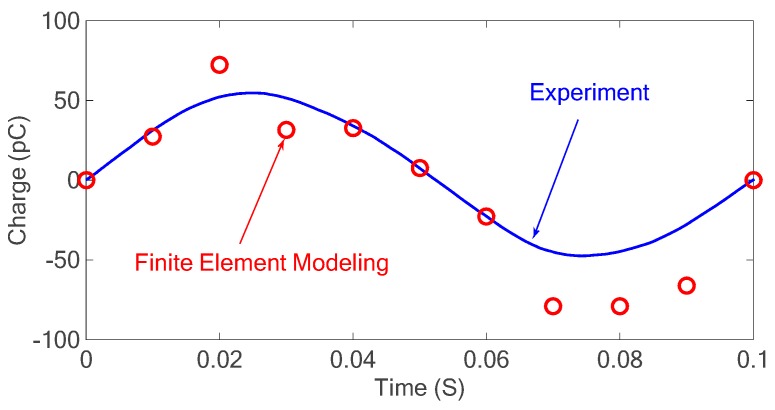
Piezoelectric results of the nanocomposite obtained from simulation and experiments.

Figure 14 shows the numerical and experimental results of the piezoelectric properties of the nanocomposite under different strains applied to the sensor. In this case, reference values from a strain gauge were used and applied as boundary conditions to the model. The resulting charge was determined from the finite element simulation and compared to the experimental results. This simulation was performed under quasi-static conditions and dynamic effects such as charge leakage and parasitic capacitance were neglected. 

The result obtained for piezoelectric coefficient in Table 2 presents a difference of 22% with respect to the experimental results. In addition, the results extracted from Figure 14 show a good agreement between the FE simulation and experimental results. Therefore, it can be concluded that the described model can predict the piezoelectric properties of the PVDF–CNT nanocomposites. The next section presents the results obtained from the experimental tests performed on the developed nanocomposite sensor and a discussion of the results.

## 4. Results and Discussion

A set of experiments was performed to validate the accuracy of the sensor developed. The FRF of a structure was obtained using the nanocomposite sensor through impulse excitation test. The response of the sensor under both static and dynamic loads were then extracted and compared with the strain gauge results as an existing commercial sensor. Finally, the piezoelectric and piezoresistive signals extracted from the nanocomposite sensor were fused to achieve an integrated sensor with higher sensitivity and accuracy compared to each signal separately.

### 4.1. Impulse Excitation and Frequency Response Function (FRF) Measurement

An impulse excitation was used for investigating the performance of the nanocomposite sensor in FRF measurement. The impulse excitation test was conducted by exciting the beam structure using a piezoelectric hammer. The resulting charge (piezoelectric response) of the nanocomposite sensor was collected along with the impact hammer signal. The FRF of the structure can be obtained by capturing the data of the applied force and the sensor response. It is important to assure that impact hammer has sufficient energy throughout the desired frequency range, and that the input energy is sufficiently transferred to the output. 

**Figure 15:**
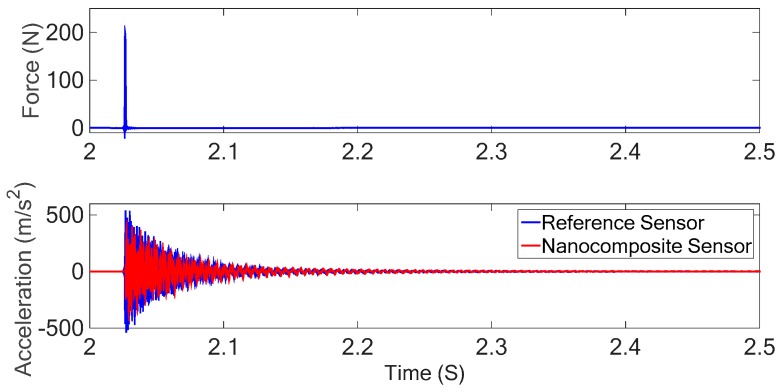
Time-domain response of the piezoelectric hammer (**top**), and nanocomposite sensor and accelerometer responses under impulse excitation (**bottom**).

**Figure 16:**
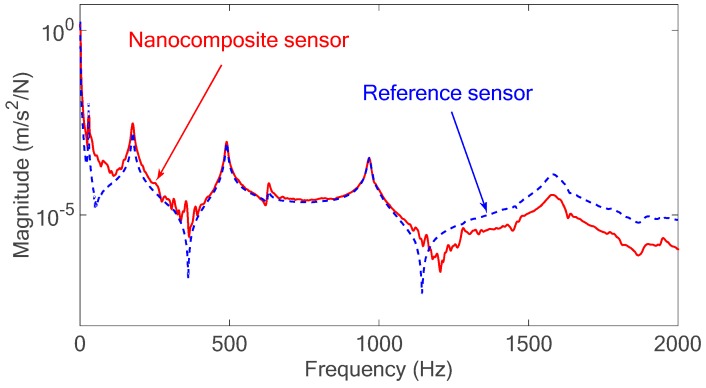
Frequency response function (FRF) of the beam structure obtained from the nanocomposite sensor measurements and Kistler accelerometer.

The time domain responses of the developed sensor are compared with the results obtained from the Kistler accelerometer as a reference sensor in Figure 15. The ratio of the response of the sensor to the applied force to the system in frequency domain results in the FRF of the structure as shown in Figure 16. The results of the nanocomposite sensor are compared with those of the accelerometer as a commercial sensor.

The FRF of the structure shows that there is a good agreement between the FRF results obtained from the nanocomposite sensor and the commercial piezoelectric sensor. Contrary to existing commercial piezoelectric sensors, the developed sensor can measure the static and low-frequency excitations as presented in next section.

### 4.2. Static and Dynamic Measurement Results

**Figure 17:**
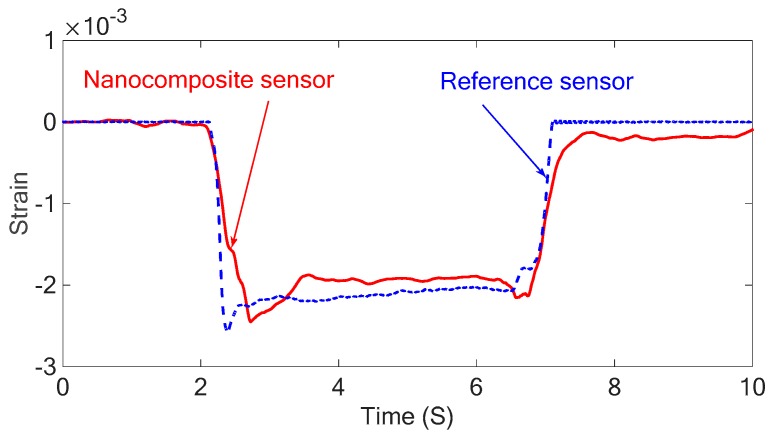
The piezoresistive response of the system under static loading obtained from the nanocomposite sensor and the metal foil strain gauge.

The piezoresistive characteristic of the sensor is only used for static measurement due to the charge leakage problem associated with the piezoelectric sensor. A static force was applied to the free end of the cantilever shown in Figure 8 and the strain of the cantilever at the sensor location was measured using the piezoresistive output of the sensor. After applying a bending load to the system, the free end of the cantilever was held for about 4 seconds at maximum displacement and returned to its original state. The results obtained from both nanocomposite sensor and the commercial strain gauge sensor are shown in Figure 17. A Savitzky–Golay filter was applied to the recorded signals in this study to minimize the effect of noise. 

The results obtained prove that there is a good agreement between the results of piezoresistive sensor and the metal foil strain gauge as reference sensor in static measurements. However, there is a small deviation between the results that can be attributed to the electric connections, i.e., the copper tape used in this study. Also, the nanocomposite sensor is attached to the beam using double-sided tape, while the strain gauge sensor is attached using super glue. Therefore, when a static force is applied to the system, the reference sensors react to this change immediately, but the difference in the bond properties of the double-sided tape does not allow the nanocomposite sensor to follow the behavior of the reference sensor accurately enough. The above results also show that the developed sensor can detect the sign of the strain. When the force is applied upward at the end of the cantilever resulting in negative strain, the resistance of the sensor decreases due to decrease of the distance between the CNT networks. Similarly, the length of the strain gauge decreases when a force is applied upward resulting in lower resistance in the sensor. The calibration factor for the piezoresistive sensor was picked $1.5\times 10^{-3}\;V^{-1}$ and this calibration constant was used for the free and forced vibration tests. 

**Figure 18:**
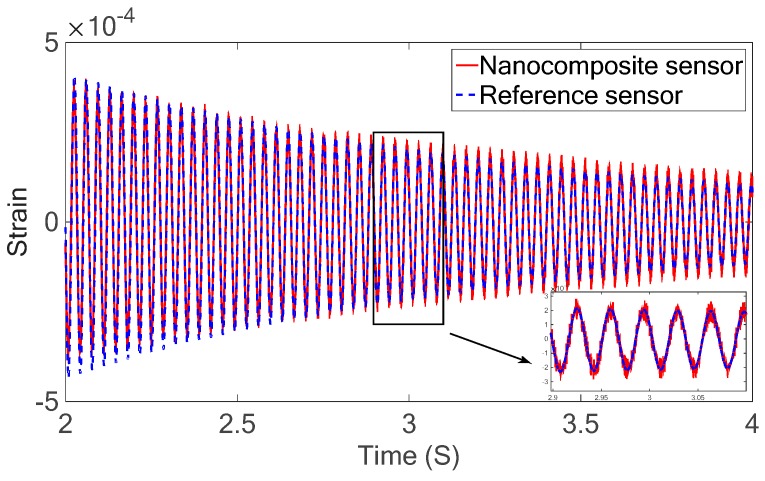
Free vibration response of the piezoelectric nanocomposite sensor and the metal foil strain gauge as reference sensor.

Dynamic excitations including the free and forced vibrations were applied to the free end of the cantilever and the strain of the beam at the sensor location was measured using the nanocomposite sensor. For the free vibration test, the beam was first pushed down 10 mm at the free end of the cantilever and then released. The results of the free vibrations for both developed sensor and the commercial strain gauge are shown in Figure 18.

The free vibration results show that there is a good agreement between the piezoelectric results and strain gauge results. For the piezoelectric results, a calibrating factor of $6.1\times 10^{-6}\;pC^{-1}$ was chosen to convert the charge output from the nanocomposite sensor to strain after matching the results obtained from both sensors. This calibration factor was used for all subsequent piezoelectric testing.

**Figure 19:**
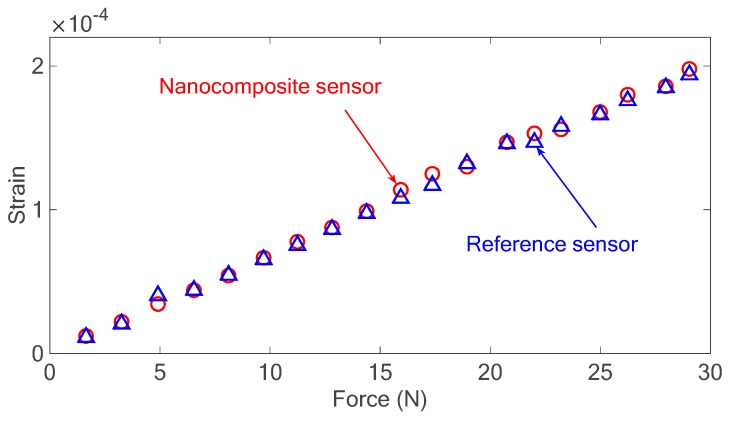
The strain of the beam under different excitation loads measured with both developed nanocomposite and metal foil strain gauge as reference sensor.

To investigate the linearity of the developed sensor, harmonic excitation was applied to the system with different amplitudes and the strain of the beam for each excitation level was measured using the nanocomposite sensor and the strain gauge as the reference sensor. The strain results were then plotted versus applied force to the system measured using a piezoelectric force sensor (Kistler 9222), as shown in Figure 19. 

The results obtained prove that there is almost a linear relationship between the applied force to the system and the resultant strain measured by the developed and reference sensors, verifying the linearity of the developed sensor. It also shows that the calibration factor is almost constant for a wide range of strains since the nanocomposite senor and the reference sensor are closely matched with each other. 

**Figure 20:**
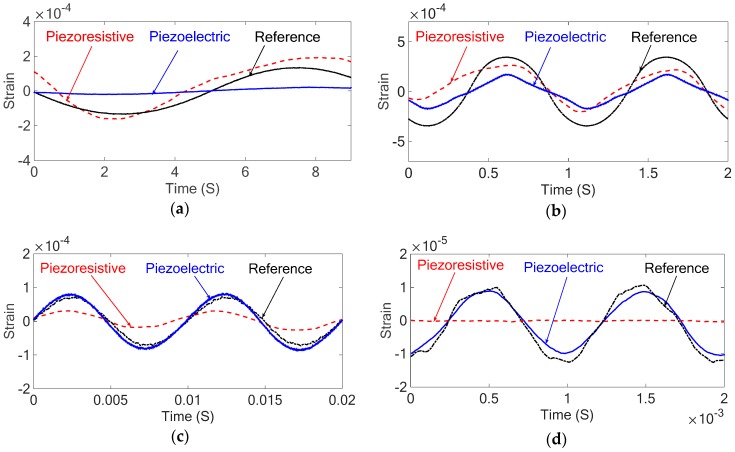
The strain of the cantilever under forced vibration at different excitation frequencies measured with piezoresistive sensor, piezoelectric sensor, and metal foil strain gauge as the reference sensor: (**a**) 0.1 Hz (**b**) 1 Hz (**c**) 100 Hz (**d**) 1000 Hz.

With both the piezoresistive and piezoelectric properties calibrated, the sensor underwent additional forced vibrations under different excitation frequencies to evaluate the performance of the nanocomposite sensor. The results of the forced vibrations with different excitation frequencies for both piezoresistive and piezoelectric responses are compared with the stain gauge as a reference sensor as shown in Figure 20.

The above results show that there is a good agreement between the forced vibration results of the piezoresistive nanocomposite sensor in low frequencies and the piezoelectric nanocomposite sensor in frequencies higher than 5 Hz, and those of the strain gauge. However, the piezoelectric sensor is not accurate in low frequencies and it can be seen in the above results that there is very small and mostly noisy response from the piezoelectric sensor at the low excitation frequency equal to 0.1 Hz. Also, the piezoresistive sensor is not capable of measuring excitations above 160 Hz and it can be seen in the above results that the piezoresistive response at 1000 Hz is predominantly noise. 

To improve the accuracy of the measured strains and increase the frequency bandwidth of the developed sensor, the piezoelectric and piezoresistive signals are combined and the fused signals are compared with those of the reference sensor in the next section.

### 4.3. Fusion of Piezoresistive and Piezoelectric Measurements

Sensor fusion techniques combine signals from different sensors to achieve more accurate results with less uncertainty compared to each signal individually. To obtain improved information of the piezoelectric and piezoresistive nanocomposite sensors, the output signals are fused, and a combined signal is achieved. 

Quite a few sensor fusion techniques have been developed in different studies using different algorithms. A method that we intend to use in this study for fusion of the piezoelectric and piezoresistive nanocomposite sensors is similar to an optimum linear smoother developed by Fraser and Potter in 1969 for the combining two independent estimates [38]. This method is based on the central limit theorem asserting that the mean of some independent random variables which are distributed identically can be approximated by normally distributed variables [39]. Based on the Fraser–Potter smoother, the fused signal is a linear combination of the two measured signals weighted by their according covariances [38,39]. Accordingly, Equation (29) was developed after modifying the Fraser–Potter smoother for fusing the piezoelectric and piezoresistive signals:

(29) $$S_{3}={\left({\left(C_{1}P_{1}\right)}^{-1}+{\left(C_{2}P_{2}\right)}^{-1}\right)}^{-1}\times \left({\left(C_{1}P_{1}\right)}^{-1}S_{1}+{\left(C_{2}P_{2}\right)}^{-1}S_{2}\right)$$

where $S_{3}$ is the fused signal, $S_{1}$ and $S_{2}$ are the piezoelectric and piezoresistive signals, $P_{1}$ and $P_{2}$ are the covariances associated with $S_{1}$ and $S_{2}$ signals, respectively, and $C_{1}$ and $C_{2}$ represent the compensation coefficients for each signal. For frequencies lower than 5 Hz, $C_{1}$ and $C_{2}$ are picked as 1 and 200, respectively, while for frequencies higher than 5 Hz these coefficients are modified to 200 and 1, respectively. The reason behind picking these coefficients is the fact that the response of the piezoresistive sensor at high frequency and piezoelectric at low frequency are very small and mostly noise, resulting in very low covariances. Therefore, the covariance of each signal needs to be weighted using the compensation coefficients, otherwise, the signal with lower variance will be the dominant based on Equation (29) and the fused signal will be more matched to the weaker signals which causes discrepancies in the fused results. 

**Figure 21:**
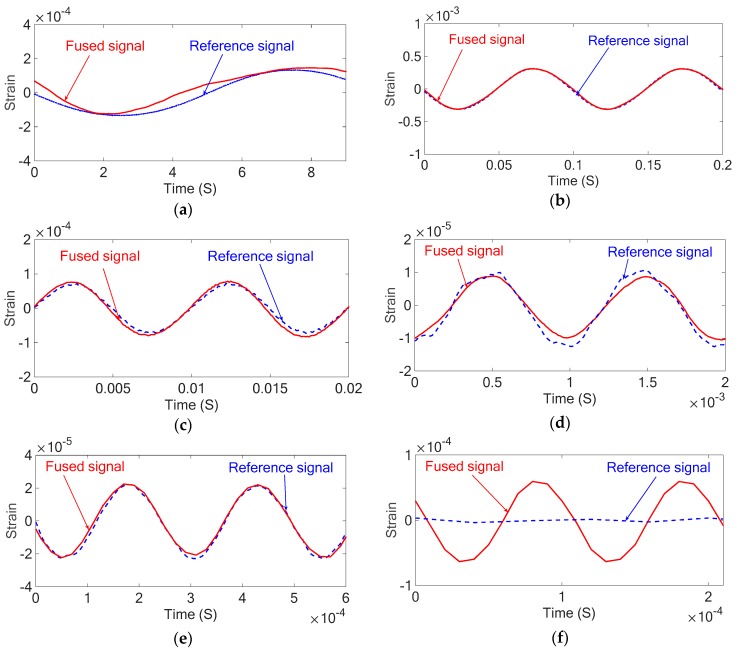
The fused and strain gauge signals for the strain of the cantilever under forced vibration at different excitation frequencies: (**a**) 0.1 Hz (**b**) 10 Hz (**c**) 100 Hz (**d**) 1 kHz (**e**) 4 kHz (**f**) 10 kHz.

The results of the dynamic tests after fusing the piezoelectric and piezoresistive properties for different excitation frequencies are shown in Figure 21.

The above results show that sensor fusion technique improves the performance and accuracy of the nanocomposite sensor; however, the very low frequencies fused signals are almost same as the piezoresistive signals as it is dominant in the sensor fusion and the high frequency signals (above 100 Hz) are almost the same as the piezoelectric signals since they are dominant. This also explains the need for the compensation coefficients to be frequency dependent. Below 5 Hz, the piezoresistive signal is weighted much higher than the piezoelectric signal and above 5 Hz, the piezoelectric signal is weighted higher. Also, it can be seen in the obtained results that at high excitation frequencies of 10 kHz, the reference sensor is no longer able to track the input while the developed sensor continues to accurately capture the signal. It shows a novel contribution of this study where the developed nanocomposite sensor is capable of measuring both low and high frequency signals while existing commercial sensors only measure either high or low frequencies.

### 4.4. Limitations and Assumptions

There are some limitations and assumptions associated with this study that might affect the performance of the developed sensor or the accuracy of the developed models. It is assumed that the sensor exhibits elastic behavior and there is no effect due to the Poisson ratio in the developed random walks model. In addition, it is considered that the β phase is the main structure of PVDF (100% beta phase), however the polymer consists of 4 different phases and this assumption might affect the numerical results. It is assumed that the used method for mixing the solution of nanoparticles and polymer provides uniform dispersion of the nanoparticles. Also, we assumed that the random dispersion of CNTs within the polymer matrix creates isotropic behaviour. Finally, the simulations were performed under quasi-static conditions and it was assumed that transient effects were negligible.

One of the main limitations observed in this study is that the effect of poling on improving the piezoelectricity decreases with increasing CNT concentration. Although it has been shown that increasing CNT concentration reduces the electrical potential required for poling to occur, it causes a reduction in the potential piezoelectric performance and reduces overall sensor sensitivity. This observation resulted in the use of different CNT concentrations to optimize the piezoelectric and piezoresistive performance, respectively. For optimal piezoelectric performance, 0.1 wt.% of CNT was used, and 2 wt.% of CNT was chosen to reach electrical percolation and optimize piezoresistive performance. The solution-mixing method used for fabricating the nanocomposite sensors contains its own challenges including imperfect dispersion of CNTs inside the polymer matrix. Other mixing techniques including melt-mixing technique might result in better performance of the nanocomposite since they provide better distribution of CNTs when the concentration of CNTs is higher. Also, adding other nanoparticles to the nanocomposite sensor might result in higher piezoelectricity and a more sensitive sensor. Finally, copper tape was used as electrodes for the sensor. For improved performance, employing other methods of depositing electrodes, such as sputtered silver, might reduce electrical contact resistance and improve the sensitivity of the developed sensor. 

The fusion method chosen may also be improved. As the sensor exhibits good piezoresistive response at low frequency with almost no piezoelectric response and the opposite occurring at high frequencies, a simple Fraser–Potter smoother may not be the most appropriate choice. The fusion algorithm is only applicable to frequency bands in which both piezoresistive and piezoelectric signals are accurate; outside of this range, and the compensation coefficients provide more of a filtering role than for fusion. In future work, a frequency-based fusion algorithm may result in further improvements.

## 5. Conclusions

A new nanocomposite-based sensor was developed in this study for strain measurements. This sensor can be used for measuring the strain of the tool and then indirect measurement of cutting forces in machining operations. The developed sensor combines piezoelectric and piezoresistive properties to achieve improved sensitivity and higher frequency bandwidth. This sensor is cost effective compared to the existing commercial sensors and is flexible and can be mounted on any surface. Two different numerical models were developed and presented for investigating the piezoelectric and piezoresistive properties of the sensor. The accuracy of these models was verified using the experimental results.

The performance of the developed sensor in strain measurement was investigated through static and dynamic experiments. The accuracy of the sensor was verified after comparing the experimental results of the sensor with those of commercial sensors. The static results showed that the developed senor can measure static forces and can detect the sign of the strain applied to the system. Also, the dynamic results showed that this sensor can be used for free and forced vibration measurements after calibration with a reference sensor. To further improve performance of the sensor, a sensor fusion algorithm was implemented. Both piezoelectric and piezoresistive signals were combined using a modified Fraser–Potter smoother approach resulting in an integrated strain sensor with improved performance. The developed sensor can measure the strains as low as 8 μstrain; however, the sensitivity and measurement range of the sensor can improve if the fabrication process is modified and the packaging process is improved. In future works, the authors would like to explore different fabrication methods along with incorporating different nanoparticles and adjusting poling conditions. Also, the effect of CNT concentration on the performance of the developed sensor will be investigated through both numerical simulations and experimental tests. Efficient electrode deposition methods and appropriate electrical connections could also improve the performance and consistency of the sensor. Improvements in performance can also be achieved with improved fusion algorithms. In addition, we are working to validate the performance of the sensor in cutting-force measurement in both boring and milling operations through in situ testing along with wireless communication.

## Figures and Tables

**Table 1:** Parameters for CNT/PVDF nanocomposites cited.

PVDF/CNT	Parameter	Unit	Value
PVDF	Elastic Modulus	GPa	8.3
	Piezoelectric coupling coefficient (*e*_13_)	cm^−2^	4.63
	Piezoelectric coupling coefficient (*e*_33_)	cm^−2^	−3.03
	Dielectric permittivity constant (*ε*_11_)	Fm^−2^	110 × 10^−12^
	Dielectric permittivity constant (*ε*_33_)	Fm^−1^	110 × 10^−12^
	Film thickness (*t*)	µm	15
CNT	Elastic modulus	GPa	700
	Dielectric permittivity constant (*ε*_33_)	Fm^−1^	120 × 10^−12^

**Table 2:** Piezoelectric charge constant results.

Parameter	Simulated	Experimental	Difference (%)
Piezoelectric charge coefficient, *d*_33_ (PcN^−1^)	24.2	31.2	22
